# Cloudy or sunny? Effects of different environmental types of urban green spaces on public physiological and psychological health under two weather conditions

**DOI:** 10.3389/fpubh.2023.1258848

**Published:** 2023-08-28

**Authors:** Saixin Cao, Zike Shang, Xi Li, Hao Luo, Lingxia Sun, Mingyan Jiang, Juan Du, Erkang Fu, Jun Ma, Nian Li, Baimeng Guo, Xiaofang Yu, Bingyang Lv, Jinde Wang

**Affiliations:** ^1^College of Landscape Architecture, Sichuan Agricultural University, Chengdu, China; ^2^Guangdong Academy of Forestry, Guangdong Provincial Key Laboratory of Silviculture Protection and Utilization, Guangzhou, China

**Keywords:** restorative environments, urban green spaces, weather conditions, environment types, environmental health

## Abstract

Numerous studies have demonstrated that urban green spaces (UGSs) benefit human health, but few have focused on the influence of weather on environmental restorativeness. This study assessed how different weather conditions and environments affect human health. We exposed 50 participants to different UGS environments under cloudy and sunny conditions and collected physiological, psychological and aesthetic preference data. The result showed that the physical and mental benefits of UGSs were stronger on sunny days (pulse: [*t* = 2.169, *p* < 0.05]; positive affect: [*Z* = −10.299, *p* < 0.001]; perceived restortiveness: [*Z* = −3.224, *p* < 0.01]). The spaces with exposed sky had greater physiological restorativeness on sunny days; the spaces with calm water had greater emotional restorativeness on cloudy days, and natural spaces with less sky exposure had greater perceived restoration in both weather conditions. The spaces with water and less sky exposure promoted psychophysiological restoration in both weather conditions. This study demonstrates that weather significantly influences the restorative potential of UGSs, and there are also restorative variations in different green space environments under two weather conditions. In future UGS planning practices, it would be advisable to select appropriate environmental types and features based on the climatic characteristics of different regions. For instance, in areas with frequent overcast conditions, incorporating serene water bodies could be advantageous, while regions with predominantly sunny weather should encompass spaces with expansive sky views. By conducting comprehensive research on restoration environments that take weather conditions into account, new insights and nature-based solutions can be provided for creating healthy human habitats in the context of global climate change.

## 1. Introduction

With rapid and intense urbanization around the world, the resulting high-density urban environments and high pressures of urban life have impacted people’s physical and mental health (1). Research has demonstrated that urban green spaces (UGSs) play an important role in human health recovery and maintenance of well-being, serving as a nature-based solution (NBS) to improve health (2, 3). In addition, weather conditions directly affect people’s perception of the environment (4) and the recovery effects of health (5). In daily life, the green space environment and weather conditions are experienced simultaneously. Interestingly, research on the individual effects of these two elements on health restoration is relatively in depth, but few studies have focused on the joint effects of the UGS environment types and weather conditions on human health. Therefore, we explored the joint effects of weather conditions and UGS environment types on physical and mental restoration to provide novel insights.

There are two prominent theories on how green space environments exert physiological and psychological restoration effects on humans: attention restoration theory (ART) and stress reduction theory (SRT). ART assumes that natural restorativeness is mainly due to the restoration of directed attention (6). In contrast, SRT emphasizes the release and reduction of stress in the environment and emphasizes emotional and physiological recovery (7). Moreover, related studies have also demonstrated that there is a positive correlation between aesthetic preference and health restoration (8–10).

Based on ART and SRT, numerous studies have indicated that green environments contribute to mitigating health deficits among residents, encompassing both psychological and physiological health restoration. Psychological health impacts involve the augmentation of positive emotions (11), reducing anxiety and stress (12), and improving perceived ability (13). Physiological health restoration encompasses the reduction of physiological parameters such as blood pressure, pulse rate, and salivary cortisol levels (14, 15), along with a decrease in the incidence and mortality rates of cardiovascular and respiratory diseases (16).

In terms of environmental characteristics, plants and water are important predictors of UGS restorativeness and preference (14, 17, 18). Even trees lacking leaves (i.e., in winter) can have positive effects on humans (19). Water bodies with good water quality and a natural form may be attractive and have restoration potential (20). Shi et al., (21) found that people prefer open spaces. In environments with rich vegetation and exposed sky, people tend to experience greater relief from depression and anxiety (22), greater perceived restoration (23) and greater physiological resilience (24); personal restoration is positively correlated with sky visibility (25). However, some scholars believe that lower sky visibility in autumn in Tokyo may improve the recovery of older individuals due to relief from heat stress and increase the vision greenery (26).

Numerous studies have demonstrated that sunny weather and good daylight could improve mood (23, 27), relieve stress (28), improve perception (29) and promote physical and mental health (30). Ulrich (31) found that daylight can affect people’s environmental preference, thereby affecting restorativeness. In contrast, severe weather affects people’s environmental preferences (32) and recovery potential (33). Research has investigated why sunny days facilitate recovery, and an in-depth physiological mechanism has been proposed. Specifically, with sunlight, ultraviolet A radiation releases nitric oxide stored in the dermis into the blood plasma, thereby dilating coronary arteries and exerting cardioprotective and antihypertensive effects (34–37).

Weather conditions have a greater impact on natural environment preferences than on artificial environment preferences (4), and severe weather has a greater impact on the restorativeness of water bodies (38). Natural environments with more green elements, more blue sky, and more sun exposure exert greater stress relief (22). Research has shown that changes in environmental quality and climatic conditions may increase attention, reduce the attractiveness of the environment, and reduce perceived compatibility and extent when visiting UGSs (39). However, regardless of the environment features, people prefer sunny days over cloudy days (40).

Although a small number of studies have focused on the impact of weather and environmental features on individual health recovery, there is still a lack of research on the restoration benefits of specific UGS environment features from a weather perspective. Therefore, we conducted comparative experiments in UGSs under both sunny and cloudy weather conditions. The physiological and psychological data of participants were measured before and after exposure to varying environmental types. Subsequently, we performed corresponding statistical analyses with the aim of addressing the following research questions: (1) Do the physiological and psychological restorativeness of UGSs remain consistent under cloudy weather conditions compared to sunny weather conditions? (2) Do different types of UGS environments have varying effects on physiological and psychological health under the two weather conditions?

## 2. Materials and methods

### 2.1. Participants

A total of 50 participants, evenly divided between males and females, were recruited for this study to control the potential confounding factors and biases arising from gender imbalance. According to the Central Limit Theorem, when the sample size exceeds 30, the sampling distribution of the mean tends to approach a normal distribution, satisfying the requirements for subsequent statistical analyses. Posters were posted within the university campus and its vicinity, and volunteers were also recruited through mobile group chat applications (WeChat) to expand the sample scope. Participants were selected then based on the following criteria: (1) self-reported normal vision and hearing, and (2) no history of cardiovascular or mental disorders. Participants were instructed to refrain from smoking, alcohol consumption, and vigorous physical activities before participating in the study. All potential participants were informed of the experimental procedures, associated risks, and provided informed consent before the commencement of the experiment. This study was approved by the Ethics Committee of the College of Landscape Architecture, Sichuan Agricultural University, China.

### 2.2. Study area, weather and sites

The study area was in Chengdu, Sichuan Province, China, which has a subtropical monsoon climate. Chengdu is located in the Sichuan Basin in the east of the Qinghai-Tibet Plateau, one of the four major basins in China. Due to the closed terrain, water vapour does not spread easily, and there are many cloudy days. The average number of annual sunshine hours in 2020 was 927.4 h, the lowest in China (41). Torshavn in the Faroe Islands, which is one of the cloudiest cities in the world, has an average annual sunshine time of only 1002.1 h (42). Thus, Chengdu is relatively representative of cloudy areas in the world.

To avoid the influence of confounding variables such as temperature, humidity, and wind speed due to the long interval between each experiment date, the experiments were conducted in autumn (November) when the cloud cover changes obviously on sunny and cloudy days and the climate is mild and pleasant for local residents (average temperature = 10–17°C). According to the American Meteorological Association, cloud cover is defined as the portion of the sky covered by clouds; on cloudy days, cloud cover is higher than 70%, and on sunny days, cloud cover is less than 20% (43). Two fully overcast days and two cloudless days were selected for the experiment based on the cloud cover reported by the China Meteorological Administration. To ensure that the temperature, humidity, and wind speed of each experiment were similar, the span of the experiment dates did not exceed 2 weeks. Objective climate data were also measured on the days of the experiment to reduce errors (Table 1). Before the experiment, the participants were informed of the temperature during the experiment and recommended to wear appropriate clothing (long-sleeved shirt and light jacket) to ensure thermal comfort.

**Table 1:** Climatic data during the experiment.

	Weather	Temperature (°C)	Relative humidity (%)	Wind speed (m/s)
Day 1 (September 3, 2021)	Cloudy	14.3 ± 1.6	75.8 ± 6.2	1.1 ± 0.4
Day 2 (September 4, 2021)	Cloudy	14.8 ± 2.1	71.8 ± 3.6	1.3 ± 0.5
Day 3 (September 9, 2021)	Sunny	13.7 ± 1.7	54.4 ± 6.1	1.4 ± 0.5
Day 4 (September 12, 2021)	Sunny	14.2 ± 1.9	62.4 ± 11.8	1.4 ± 0.7

Values shown are the mean ± SD.

**Figure 1:**
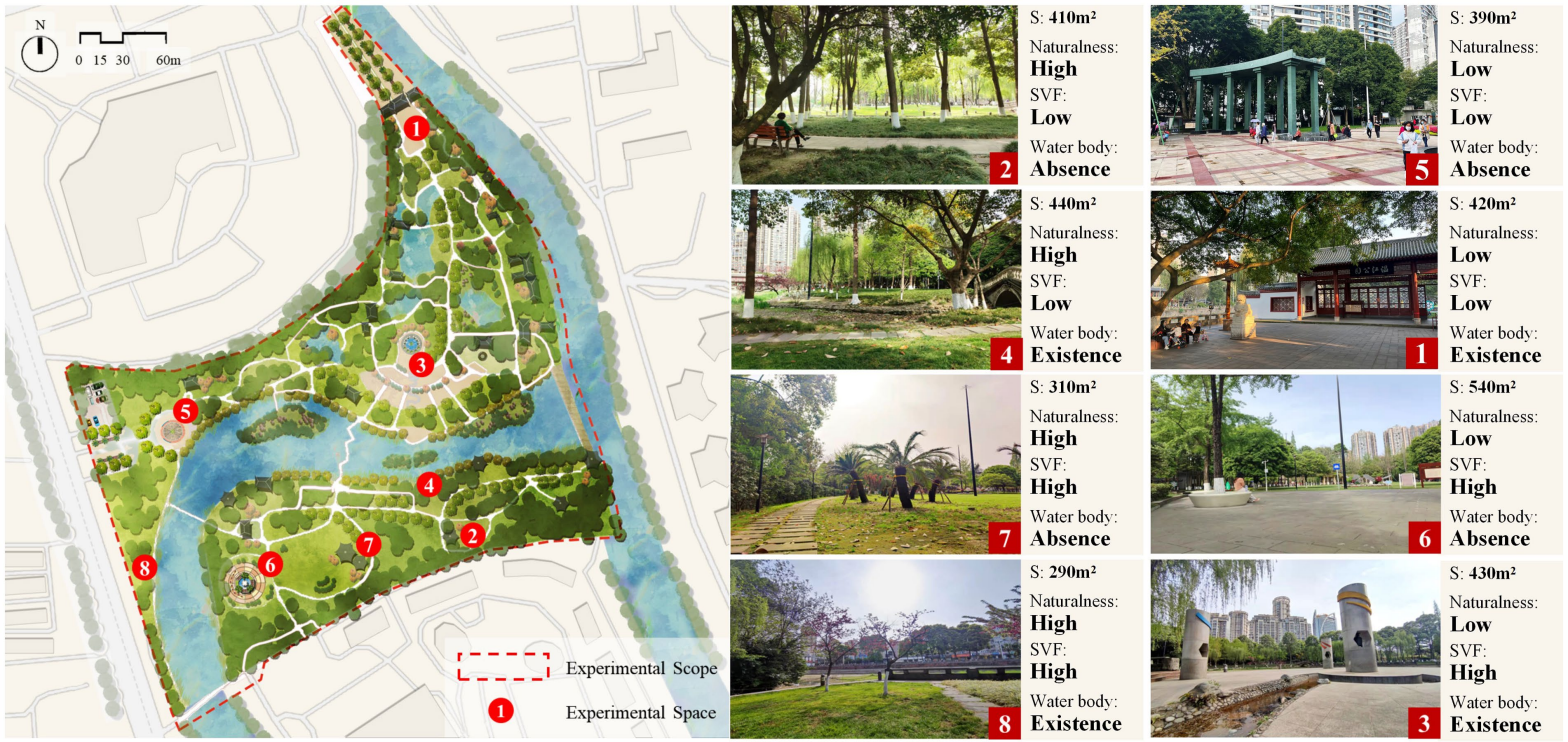
Descriptions and photograph of the 8 study spaces.

The experimental site was Wenjiang Park in Wenjiang District, Chengdu. This park is the largest and most visited park in the area. Based on previous studies on environment characteristics, we extracted three characteristic dimensions of UGSs closely related to weather conditions and health: naturalness, water body, and the sky view factor (SVF, the fraction of visible sky in a specific place). Previous studies have shown that weather has a greater impact on the restorativeness of natural environment than on the restorativeness of artificial environments (4, 40), that environment restorative potential of bodies of water are highly influenced by weather (38), and that the SVF directly affects people’s perceptions of weather conditions and the restorativeness of the environment (24). Three environmental characteristic dimensions were then classified: naturalness (high vs. low), body of water (presence vs. absence), and SVF (high vs. low). Naturalness was assessed by taking panoramic photos of eight experimental spaces from the participant’s view and then calculating the coverage density of natural components (plants, water, and topography) and artificial components (landscape constructions, roads and pavement, and garden facilities) in each photo (44, 45). When the density of natural components in a space was higher than that of artificial components, the space was considered to have high naturalness; otherwise, it was considered to have low naturalness. The SVF was evaluated by photographing the sky using a fish-eye camera lens (Nikon D750 digital camera; Nikon AF DX Fisheye-NIKKOR 10.5 mm f/2.8G ED) at human height (46). When the proportion of visible sky was higher than 50%, the SVF was considered high; otherwise, it was considered low. The body of water was evaluated by the presence or absence of water in the sight range inside and around the experimental spot. After combining the 3 environmental features, 8 environmental types were generated. The corresponding 8 spaces in Wenjiang Park were selected for experiments after the field investigation (Figure 1). See supplementary material (Supplementary Figure S1) for the specific environmental information of the 8 spaces.

### 2.3. Procedure

The 50 participants were randomly divided into two groups, and each group was tested on a cloudy day and a sunny day, and the two experiments were separated by a week to eliminate legacy effects. The experiment was completed over a total of 4 days and within 2 weeks. Each day’s experiment involved 25 participants, who were divided into 5 groups of 5. Experiments were conducted at the same times (10:00–12:00 and 13:30–15:30) each day to eliminate the influence of diurnal changes in physiological rhythms. Each group started the experiment at a different space and switched spaces in sequence to eliminate order effects. To eliminate fatigue, each group visited 4 spaces in the morning and visited the other 4 spaces in the afternoon. To eliminate legacy effects, the participants closed their eyes and rested for 3 min after each switch of space (before starting a new experiment).

**Figure 2:**
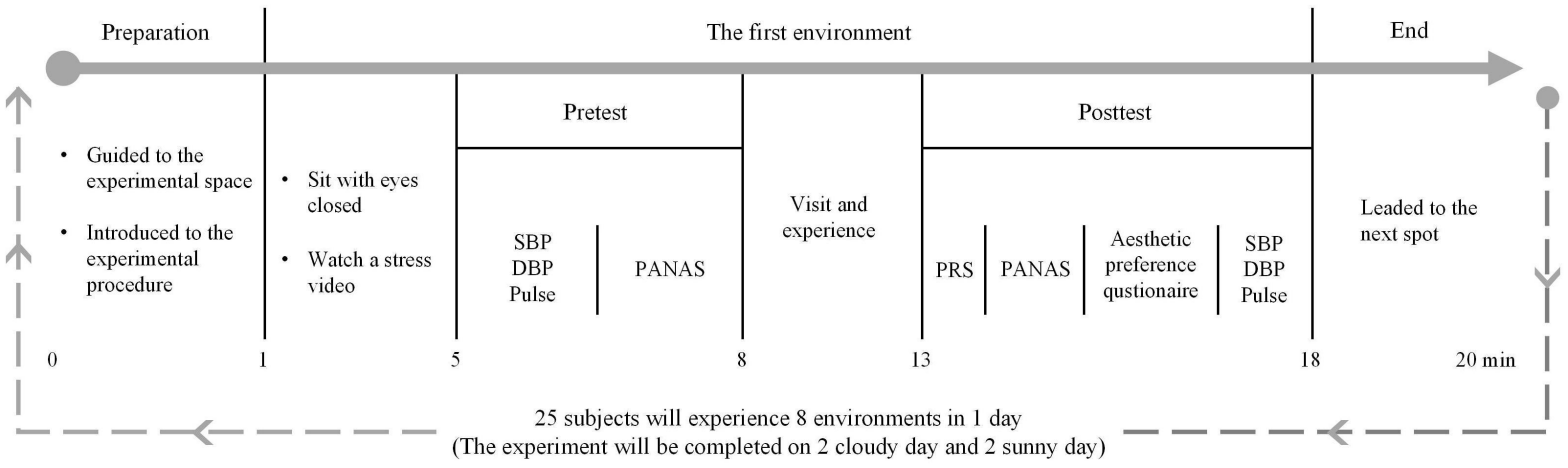
Experimental flowchart.

The participants were first guided to the designated site by the researchers, who introduced the experimental process. After arriving at the experimental site, the participants closed their eyes and sat for 3 min to achieve a state of calm (47). After reaching a calm state, they were exposed to a stressful video for 1 min. The experimenters closely monitored the participants’ emotional states throughout and ensured their right to withdraw from the stress video at any time (In the four-day experiment, only one female participant raised an issue regarding the slightly high volume of the stress video; subsequently, we lowered the volume and completed the entire experiment). Simultaneously, a counselor was available throughout the experiment to provide potential psychological support as needed, to ensure the well-being of the participants. Participants then took physiological measurements and completed positive and negative affect schedule (PANAS). Ulrich (48) found obvious physical and mental recovery after 3–5 min of contact with the natural environment; thus, after completing the questionnaire, the participants experienced in the experimental area for approximately 5 min. They were allowed to walk and sit but were instructed not to talk to others or look at electronic devices. After the visiting experience, the participants completed the perceived restorativeness scale (PRS) and PANAS and answered two questions about their aesthetic preferences. The researchers then measured and recorded their physiological indicators again. We mitigated the discomfort caused by the constriction of the blood pressure cuff by maximizing the interval between the two blood pressure measurements. After completing a space experiment, researchers led participants to the next space; and the same process was repeated at each space (Figure 2). The demographic characteristics of the participants were collected in advance of the experiment.

### 2.4. Measurements

#### 2.4.1. Physiological measures

Blood pressure is a crucial indicator of physical health status, capable of reflecting states of alertness or relaxation. Simultaneously, blood pressure measurement possesses the advantages of high portability, meeting outdoor experimental demands, and imposing a smaller burden on participants. Therefore, blood pressure was ultimately selected as the physiological measurement index for restoration. In the experiment, systolic blood pressure (SBP; mmHg), diastolic blood pressure (DBP; mmHg), and pulse (bpm) were measured on the subjects’ left arm using a portable electronic blood pressure monitor (Omron, HEM-7011, China). Physiological measurements were conducted both before and after each spatial experience.

#### 2.4.2. Psychological measures

Based on ART and SRT, the association between UGS and psychological health primarily pertains to perceived restoration and emotional recovery. For emotional recovery, we selected the Positive and Negative Affect Schedule (PANAS), a well-established scale for measuring positive affect (PA) and negative affect (NA), which has been comprehensively documented for its reliability and validity (49). The PANAS measures positive affect (PA) and negative affect (NA) using 10 items each (a total of 20 items). The scale ranges from 1 (very slightly) to 5 (extremely) to rate the subjective feelings of the participants, with higher scores representing stronger emotional experiences. We conducted PANAS assessments before and after each space experience (Supplementary Table S1).

In terms of perceived restoration, the currently predominant measurement method is the Perceived Restorative Scale (PRS). It assesses the quality of environment restorativeness along 4 characteristic dimensions in ART (16): being away (i.e., escaping from the routine aspects of one’s life), fascination (a scene or object interesting enough to hold one’s attention), compatibility (match between an individual’s needs or desires and what the environment offers) and extent (there are various exhibits and displays to explore in this setting). To prevent participant fatigue, a short version of the revised PRS by Hartig et al. (50) and Huang et al. (51) was selected; it has a total of 18 items (Supplementary Table S2).

Two aesthetic preference questions were used to assess the participants’ aesthetic preferences for the current sky and environment. To avoid ambiguity, the sky aesthetic preference in the questionnaire was defined as “the sky is beautiful enough to attract people,” and the environmental aesthetic preference was defined as “the scenery is beautiful enough to attract people.” The two specific questions were as follows: (1) “Look at the sky. How would you rate the beauty of the sky at this moment?” and (2) “Look around. How would you rate the beauty of the environment at this moment?” These items were scored on a 5-point Likert scale (Supplementary Table S3).

### 2.5. Statistical analysis

The Mann–Whitney U test and Kruskal–Wallis test were used to analyse the demographic characteristics of participants. A paired t test was used to analyse physiological data. The Wilcoxon signed-rank test and Kruskal–Wallis test were used to analyse aesthetic preferences and psychological data. The statistical analysis was performed with SPSS 27.0 (SPSS Inc., Chicago, IL, United States), and a *p* <0.05 was considered to indicate statistical significance.

## 3. Results

### 3.1. Demographic characteristics

**Figure 3:**
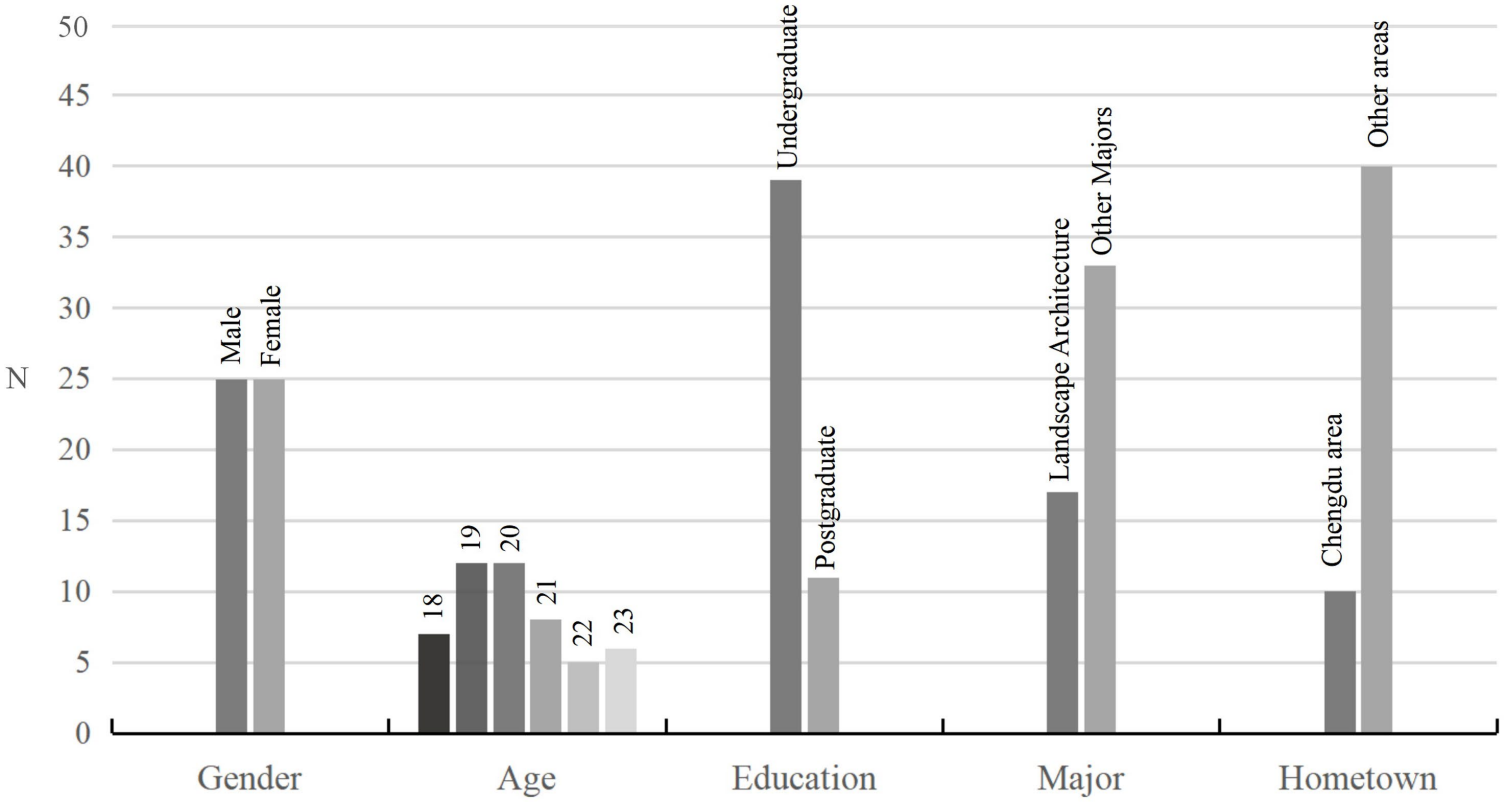
Demographic characteristics of participants.

The analysis of demographic characteristics is shown in Figure 3. There were equal numbers of male and female participants in this experiment, and almost half of all participants were aged 19 or 20 years. In terms of educational background, undergraduate participants accounted for more than 70% of the sample, and there were half as many participants majoring in landscape architecture as participants with a different major. Regarding region, 20% of the participants were from Chengdu, and 80% were from other regions.

As shown in Table 2, sex had a significant impact on the aesthetic preference and psychological restorativeness of UGSs. Women had a stronger aesthetic preference under the sunny condition (*Z* = −2.699, *p* < 0.01), and UGSs had greater psychological restorativeness for women under both weather condition (sunny [*Z* = −3.559, *p* < 0.001]; cloudy [*Z* = −3.176, *p* < 0.01]). Compared with participants from other regions, participants from the Chengdu region had a significantly lower aesthetic preference for UGSs on cloudy days (*Z* = −2.060, *p* < 0.05).

**Table 2:** The relationships among demographic characteristics, aesthetic preferences and perceived restoration.

Demographic characteristics (*N* = 50)		Aesthetic preference (Cloudy)		Aesthetic preference (Sunny)		Perceived restoration (Cloudy)		Perceived restoration (Sunny)	
		Mean rank	*p*	Mean rank	*p*	Mean rank	*p*	Mean rank	*p*
Sex	Male	199.22	0.812	185.90	0.007**	182.15	0.001**	179.93	0.000***
	Female	201.79		215.10		218.86		221.07	
Age	18	213.50	0.826	185.13	0.411	182.20	0.714	184.29	0.234
	19	190.36		216.73		208.10		208.44	
	20	201.72		193.92		196.41		181.87	
	21	195.78		193.30		213.02		223.59	
	22	199.81		192.63		205.36		202.59	
	23	210.04		215.29		194.09		208.27	
Education	Undergraduate	195.28	0.069	201.18	0.814	206.64	0.052	199.29	0.692
	Postgraduate	219.02		198.10		181.74		204.81	
Major	Landscape architecture	204.96	0.558	203.35	0.708	199.26	0.879	214.14	0.094
	Other	198.25		199.06		201.13		193.63	
Hometown	Chengdu	178.41	0.039*	209.74	0.389	199.26	0.052	213.22	0.267
	Other region	206.11		198.15		201.13		197.27	

*p < 0.05, **p < 0.01, ***p < 0.001.

### 3.2. Aesthetic preference

The Wilcoxon signed-rank test showed (Table 3) that subjects’ aesthetic preferences for the sky and environment on sunny days were significantly higher than those on cloudy days (sky [*Z* = −11.46, *p* < 0.001]; environment [*Z* = − 4.42, *p* < 0.001]).

**Table 3:** Sky aesthetic preference and environment aesthetic preference under two weather conditions.

Variable		*N*	Median (IQR)	*Z*	*p*
Sky aesthetic preference	Cloudy	400	2.00 (2.00–3.00)	−11.46	0.000***
	Sunny	400	4.00 (2.00–4.00)		
Environment aesthetic preference	Cloudy	400	3.00 (3.00–4.00)	−4.42	0.000***
	Sunny	400	4.00 (3.00–4.00)		

*p < 0.05, **p < 0.01, ***p < 0.001. IQR, interquartile range.

The Kruskal–Wallis test revealed significant differences in the mean aesthetic ratings among the 8 spaces under the two weather conditions (cloudy [*H* = 32.357, *df* = 7, *p* < 0.001]; sunny [*H* = 27.622, *df* = 7, *p* < 0.001]). Under the cloudy condition, spaces 2, 3, and 4 had higher aesthetic preference scores, and space 4 had the highest score (Table 4A). Under sunny conditions, spaces 2, 3, and 4 had higher aesthetic preference scores, and space 2 had the highest score (Table 4B).

**Table 4:** Aesthetic preferences of 8 environments types in two weather conditions (*N* = 50).

A. Cloudy condition								
	Space 1	Space 2	Space 3	Space 4	Space 5	Space 6	Space 7	Space 8
Median (IQR)	3 (3–4)	4 (3–4)	4 (3–4)	4 (3–4)	3 (3–4)	3 (3–4)	3 (3–4)	3 (3–4)
Mean rank	159.24	230.47	221.51	256.79	188.19	170.02	188.98	188.8
Ranking	8	2	3	1	6	7	4	5
B. Sunny condition								
Median (IQR)	3 (3–4)	4 (3–5)	4 (3–4)	4 (3–4.25)	3 (3–4)	4 (3–4)	4 (3–4)	3 (3–4)
Mean rank	155.1	243.75	220.28	223.95	170.95	207.79	206.80	175.38
Ranking	8	1	3	2	7	4	5	6

IQR, interquartile range. A rank has been awarded to each space based on the mean rank.

### 3.3. Physiological results

The physiological indicators suggested that participants were more relaxed after visiting the 8 UGSs on sunny days than on cloudy days (Table 5). The paired t test showed that the pulse of participants after the UGS experience was significantly lower in the sunny condition than that in the cloudy condition (72.97 ± 10.89 and 74.13 ± 10.20, respectively; *t* = 2.169, *p* < 0.05). Participants’ SBP and DBP did not significantly differ between sunny days and cloudy days (SBP [119.04 ± 13.30 and 119.92 ± 12.17, *t* = 1.536, *p* = 0.125]; DBP [76.32 ± 9.00 and 76.87 ± 9.11, *t* = 1.292, *p* = 0.197]).

**Table 5:** Comparison of SBP, DBP, and pulse after experiencing UGSs under the two weather conditions.

Variable		*N*	M	SD	*t*	*p*
SBP	Cloudy	400	119.92	12.17	1.536	0.125
	Sunny	400	119.04	13.30		
DBP	Cloudy	400	76.87	9.11	1.292	0.197
	Sunny	400	76.32	9.00		
Pulse	Cloudy	400	74.13	11.20	2.169	0.031*
	Sunny	400	72.97	10.89		

*p < 0.05, **p < 0.01, ***p < 0.001. M, mean value; SD, standard deviation.

**Figure 4:**
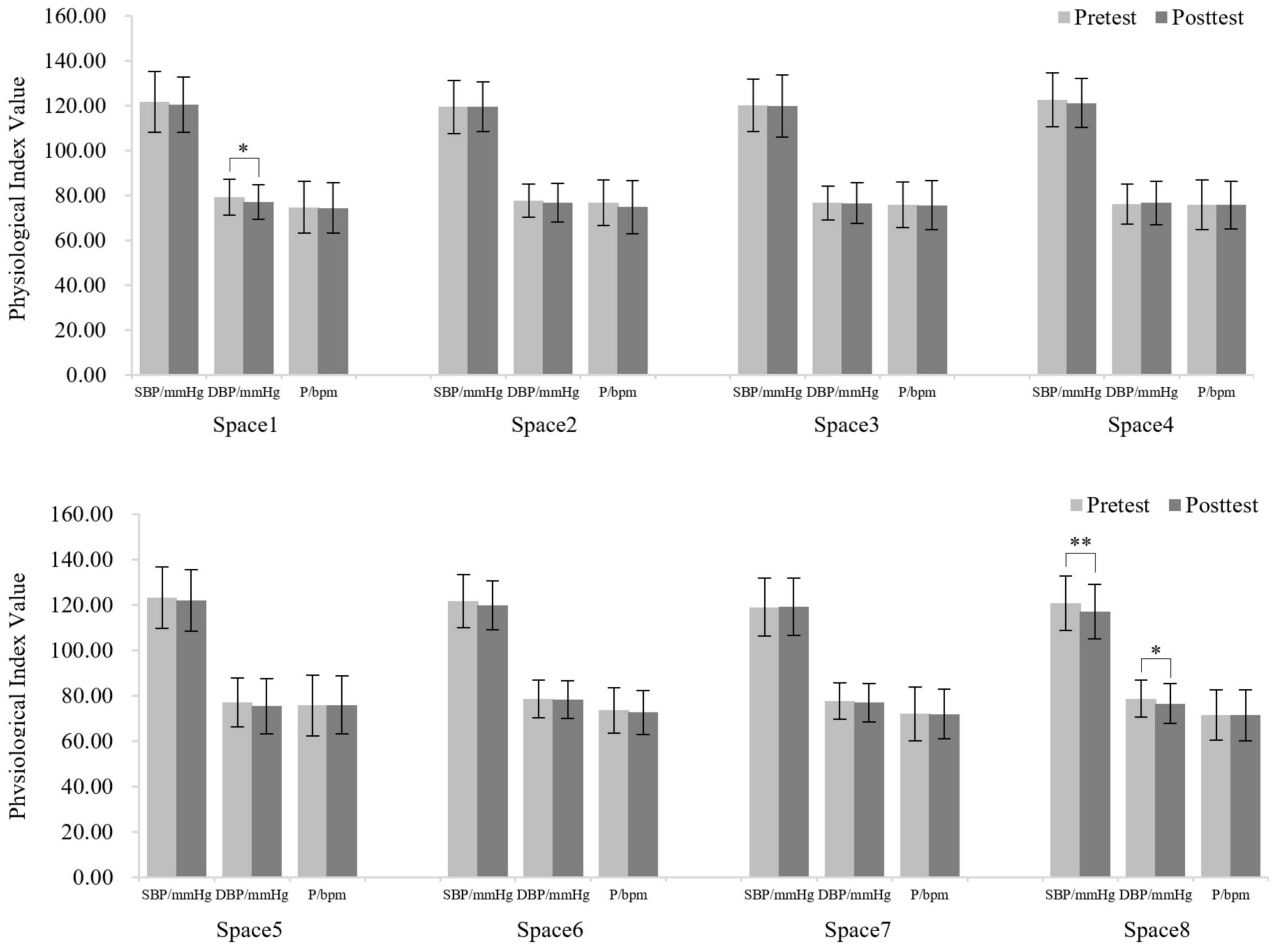
Blood pressure and pulse values before and after visiting the 8 spaces on cloudy days. *N* = 50. Values shown are the mean ± SD. *p < 0.05, **p < 0.01.

As shown in Figure 4, participants’ SBP, DBP and pulse decreased after visiting the 8 spaces on cloudy days; however, only 2 spaces produced a significant decrease in these physiological indicators. SBP (120.70 ± 11.99 and 117.16 ± 11.93, *t* = 3.87, *df* = 49, *p* < 0.01) and DBP (78.80 ± 8.07 and 76.66 ± 8.72, *t* = 2.46, *df* = 49, *p* < 0.05) were significantly decreased after visiting space 8. DBP (79.26 ± 8.10 and 77.20 ± 7.66, *t* = 2.07, *df* = 49, *p* < 0.05) was significantly decreased after visiting space 1.

**Figure 5:**
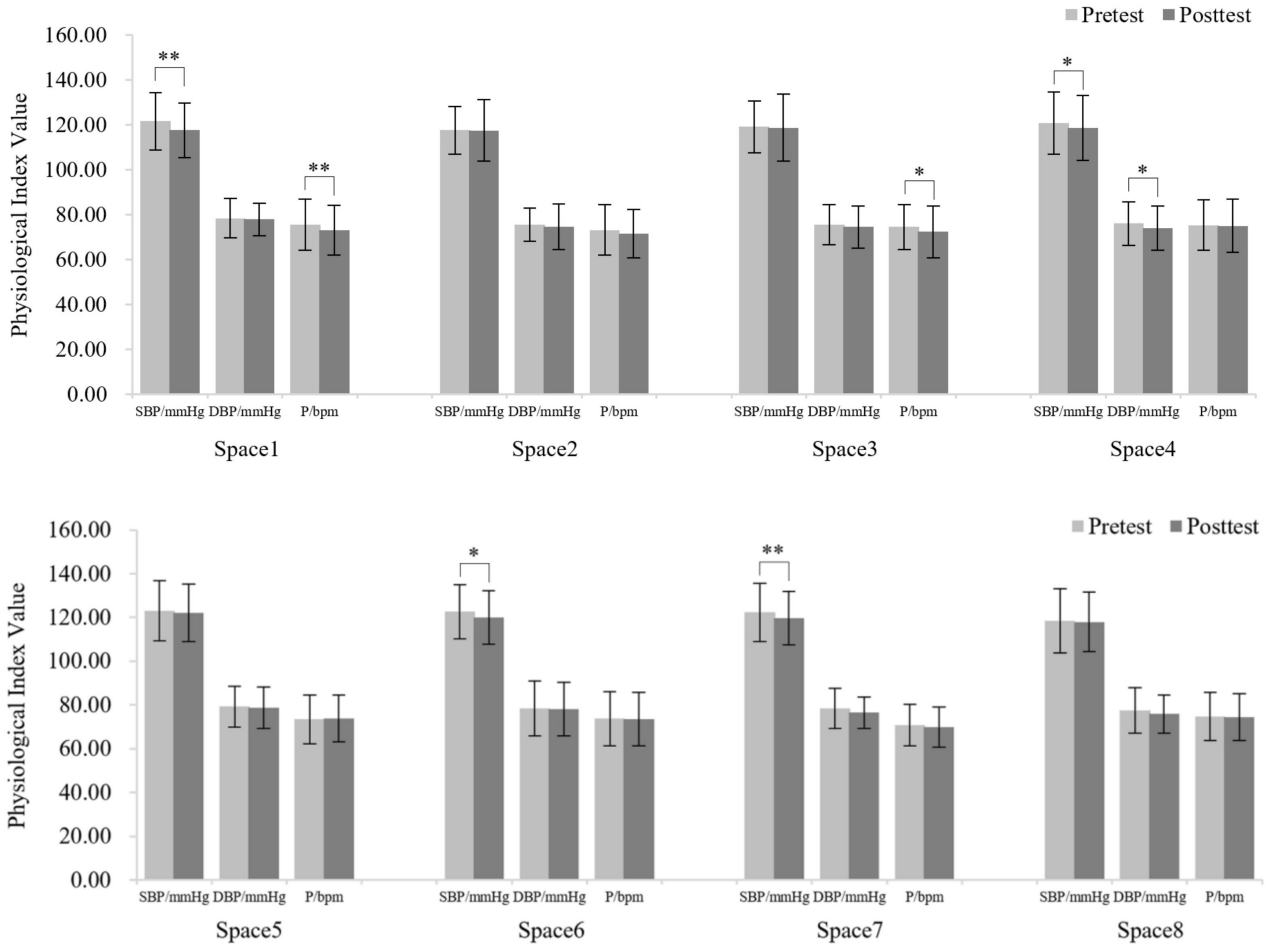
Blood pressure and pulse values before and after visiting the 8 spaces on sunny days. *N* = 50. Values shown are the mean ± SD. *p < 0.05, **p < 0.01.

As shown in Figure 5, participants’ physiological indicators decreased after visiting the 8 spaces on sunny days, and 5 spaces produced a significant decrease in these indicators. Specifically, SBP (121.58 ± 12.69 and 117.57 ± 12.12, *t* = 3.86, *df* = 49, *p* < 0.01) and pulse (75.50 ± 11.30 and 73.16 ± 11.11, *t* = 2.75, *df* = 49, *p* < 0.01) were extremely significantly decreased after visiting space 1. SBP (120.70 ± 13.87 and 118.52 ± 14.48, *t* = 2.09, *df* = 49, *p* < 0.05) and DBP (76.02 ± 9.74 and 74.06 ± 9.89, *t* = 2.26, *df* = 49, *p* < 0.05) were significantly decreased after visiting at space 4. SBP (122.40 ± 13.24 and 119.68 ± 12.15, *t* = 2.83, *df* = 49, *p* < 0.01) decreased significantly after visiting space 7; pulse (74.50 ± 9.90 and 72.32 ± 11.45, *t* = 2.33, *df* = 49, *p* < 0.05) decreased significantly after visiting space 3, and SBP (122.72 ± 12.43 and 120.14 ± 12.19, *t* = 2.38, *df* = 49, *p* < 0.05) was significantly decreased after visiting space 6.

### 3.4. Psychological results

#### 3.4.1. Emotional recovery

After visiting the 8 spaces in two weather conditions, participants exhibited stronger emotional restoration on sunny days. The Wilcoxon signed-rank test (Table 6) showed that the participants’ PA scores after visiting UGSs under the sunny condition were significantly higher than those under the cloudy condition (Z = −10.299, *p* < 0.001). There were no significant differences in NA scores between sunny and cloudy days (Z = −0.861, *p* = 0.389).

**Table 6:** Comparison of PA and NA scores after visiting UGSs under the two weather conditions.

Variable		*N*	Median (IQR)	*Z*	*p*
PA score	Cloudy	400	23.00 (17.25, 28.00)	−10.299	0.000***
	Sunny	400	27.00 (20.25, 33.00)		
NA score	Cloudy	400	11.00 (10.00, 14.00)	−0.861	0.389
	Sunny	400	11.00 (10.00, 14.00)		

*p < 0.05, **p < 0.01, ***p < 0.001. IQR, interquartile range.

**Figure 6:**
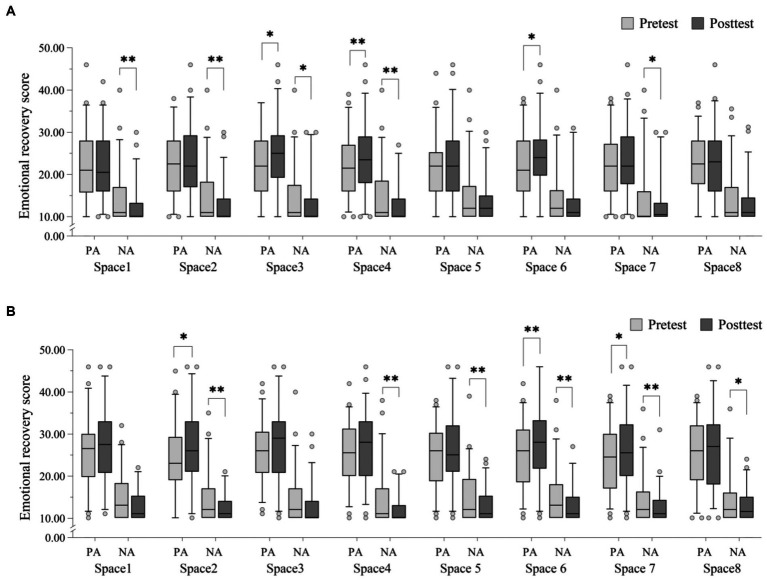
PANAS subscale scores before and after visiting 8 spaces under the two weather conditions. **(A)** Cloudy condition; **(B)** sunny condition. *N* = 50. Data shown are the median (central line), interquartile range (box margins), adjacent values (whiskers), and outliers (dots). *p < 0.05, **p < 0.01.

As shown in Figure 6A, after visiting spaces 3 and 4 under the cloudy condition, PA scores were significantly increased, and NA scores were significantly decreased. PA scores significantly increased after visiting space 6, and NA scores significantly decreased after visiting spaces 1, 2, and 7. As shown in Figure 6B, PA scores increased and NA scores decreased after visiting the 8 spaces under the sunny condition. After visiting spaces 2, 6, and 7, PA scores were significantly increased, and NA scores were significantly decreased. NA scores decreased significantly after visiting spaces 4, 5 and 8.

#### 3.4.2. Perceived restoration

The Wilcoxon signed-rank test (Table 7) showed that the scores of each dimension of perceived restoration (being away [*Z* = −2.059, *p* < 0.05], extent [*Z* = −3.290, *p* < 0.01], fascination [*Z* = −3.291, *p* < 0.01], and compatibility [*Z* = −2.280, *p* < 0.01]) and the overall restorative potential (*Z* = −3.224, *p* < 0.01) of spaces were significantly higher on sunny days than on cloudy days.

**Table 7:** Comparison of perceived restoration scores under the two weather conditions.

Variable		*N*	Median (IQR)	*Z*	*p*
Being away	Cloudy	400	4.40 (3.60–5.20)	−2.059	0.040*
	Sunny	400	4.40 (3.80–5.40)		
Extent	Cloudy	400	4.50 (3.75–5.25)	−3.290	0.001**
	Sunny	400	4.75 (4.00–5.50)		
Fascination	Cloudy	400	4.25 (3.50–5.00)	−3.291	0.001**
	Sunny	400	4.63 (4.00–5.50)		
Compatibility	Cloudy	400	4.40 (3.60–5.15)	−2.280	0.005**
	Sunny	400	4.60 (4.00–5.40)		
Overall restorative potential	Cloudy	400	4.34 (3.68–5.11)	−3.224	0.001**
	Sunny	400	4.58 (4.00–5.28)		

*p < 0.05, **p < 0.01, ***p < 0.001. IQR, interquartile range.

The PRS scores of each space under the cloudy condition are shown in Table 8A. The Kruskal–Wallis test demonstrated that there were significant differences in the “being away” (*H* = 25.893, *p* < 0.01) and overall restorative potential (*H* = 14.820, *p* = 0.038) among the 8 spaces, but there were no significant differences in other dimensions. Overall, spaces 2 and 4 had a positive effect on perceived restoration under the cloudy condition.

**Table 8:** Perceived restoration effect of different UGSs under the two weather conditions (*N* = 50).

A. Cloudy condition									
		Space 1	Space 2	Space 3	Space 4	Space 5	Space 6	Space 7	Space 8
Being away	Mean rank	161.26	254.86	182.86	227.19	186.07	169.84	211.27	210.65
	Ranking	8	1	6	2	5	7	3	4
Extent	Mean rank	181.83	222.31	198.47	228.59	183.89	185.46	197.69	205.76
	Ranking	8	2	4	1	7	6	5	3
Fascination	Mean rank	179.29	224.13	207.98	232.04	180.36	187.91	190.55	201.74
	Ranking	8	2	3	1	7	6	5	4
Compatibility	Mean rank	168.48	235.63	203.30	228.53	188.90	179.06	198.92	201.18
	Ranking	8	1	3	2	6	7	5	4
Overall restoration	Mean rank	170.32	237.37	197.00	230.79	184.08	179.17	198.99	206.28
	Ranking	8	1	5	2	6	7	4	3
B. Sunny condition									
Being away	Mean rank	151.98	259.74	168.14	221.94	200.11	171.5	220.62	209.97
	Ranking	8	1	7	2	5	6	3	4
Extent	Mean rank	170.02	223.67	214.04	219.86	182.24	188.95	203.55	201.67
	Ranking	8	1	3	2	7	6	4	5
Fascination	Mean rank	168.08	219.57	226.65	229.12	168.85	183.54	202.36	205.83
	Ranking	8	3	2	1	7	6	5	4
Compatibility	Mean rank	164.81	219.82	194.29	221.46	184.42	190.83	210.62	217.75
	Ranking	8	2	5	1	7	6	4	3
Overall restoration	Mean rank	159.18	234.22	199.58	224.55	182.47	182.43	210.06	211.51
	Ranking	8	1	5	2	6	7	4	3

Verified by the Kruskal–Wallis test. A rank was awarded to each space based on the mean rank.

The PRS scores of each space under the sunny condition are shown in Table 8B. The Kruskal–Wallis test demonstrated that there were significant differences in “being away” (*H* = 32.677, *p* < 0.001), fascination (*H* = 15.945, *p* = 0.026) and overall restorative potential (*H* = 16.041, *p* = 0.025) among the 8 spaces, but there were no significant differences in other dimensions. Overall, spaces 2 and 4 had a positive effect on perceived restoration under the sunny condition.

## 4. Discussion

### 4.1. Demographic characteristics

Previous studies have shown that women have stronger aesthetic preferences for green spaces (52). However, we did not obtain similar results under cloudy conditions. Lis et al. demonstrated that perceived danger plays a significant mediating role in the process where the environment influences preference in natural settings (53). Generally, women are more sensitive to their surroundings (54) and are more concerned about the safety of dimly lit environments (55). Cloudy weather with poor sunlight may trigger nocturnal fears (38), and thereby influence women’s aesthetic preferences for UGSs under overcast conditions.

Under both weather conditions, women’s perceived restoration potential was stronger than that of men. This supports previous findings that women experience better stress recovery (56), vitality (57), and emotional improvement (58) in UGSs. Our study further confirms that UGSs can better promote women’s perceived restoration. This may be attributed to women’s lower self-reported vitality in daily life (57) and their greater awareness of the health benefits of natural environments (56), making them more in need of restorative environments and more receptive to the benefits of nature.

In addition, the results showed that compared with subjects from other regions, those from the Chengdu area were less pleased with the cloudy condition. von Lindern (59) proved that the weaker the perceived setting interdependencies between the environment and crowds, the stronger the sense of being away and the restorativeness of the environment. Considering that there are significantly more cloudy days in Chengdu than in other regions, subjects from Chengdu will have a weaker sense of being away on cloudy days; thus, they have a lower preference for UGSs on cloudy days.

### 4.2. Aesthetic preference

The results showed that people prefer sunny sky and that the aesthetic ratings of each space were higher on sunny days than on cloudy days. Beute and de Kort (4) proposed that sunny days are more likely to trigger positive associations (with summer, weekends, or holidays) than cloudy days (*F* = 30.9, *p* < 0.001); this may explain why people preferred sunny sky in the present study.

We further compared the preference scores of each space under the two weather conditions and found that spaces 2 and 4 (which had high naturalness and low SVF) had higher aesthetic ratings on cloudy and sunny days, with ratings higher than those of spaces 1 and 5 (which had low naturalness and low SVF). Naturalness was the measured environment feature that differed between the two types of environments, further indicating that people have a stronger aesthetic preference for natural environments (17). This may be because individuals are more likely to approach nature in natural environments than in artificial environments. According to biophilia theory (60), humans have an innate impulse to approach and connect with nature; thus, an environment with higher naturalness may evoke stronger aesthetic preferences in both weathers.

### 4.3. Physiological relaxation

The results showed that the physiological restoration benefits of UGSs were enhanced on sunny days compared to cloudy days. Previous studies have demonstrated that sunlight has cardioprotective and antihypertensive effects (35–37). Our study tested this notion and further found that sunlight exposure led to a significant decrease in pulse, resulting in greater physiological recovery when visiting UGSs on sunny days.

We found that spaces with water bodies improved physiological recovery under both weather conditions (cloudy [spaces 1 and 8]; sunny [spaces 1, 3, and 4]). This may be because water bodies make it easier for people to achieve a state of calm and relaxation by improving the microclimate (38). We also found that spaces with high SVF (space 3, 6, and 7) led to greater physiological recovery on sunny days than on cloudy days. Benita and Tunçer (24) found that UGSs with higher sky exposure tended to reduce physiological stress. Our study supports this result and further demonstrated that the physiological restoration effect of such environments is stronger on sunny days. One possible explanation is that a higher SVF increases exposure to sunlight on sunny days, and exposure to sufficient sunlight reduces physiological stress through underlying physiological mechanisms (61). Additionally, a higher SVF indicates an increased proportion of sky in the field of view. Due to the positive correlation between aesthetic preference and restoration benefits (18, 62), people’s preference for blue sky on sunny days mediates the recovery benefits of this environment type.

### 4.4. Psychological restoration

The results showed that people’s positive emotions and perceived restoration after visiting UGS were better on sunny days than on cloudy days. This supports the findings of Ulrich’s study that positive responses to nature may be enhanced by sunlight due to long-term evolutionary adaptations to sunlight (31). For example, a sunny environment is associated with less danger and less psychological stress and tension than an overcast environment. Different weather conditions provide different emotional cues; sunny days are often associated with more positive emotions (63, 64). Therefore, UGSs may have better psychological restoration effects on sunny days. NA scores were not significantly reduced in this study, which is consistent with a meta-analysis by McMahan and Estes (11), which found that PA scores increased (dependent on condition) but NA scores did not (*Q* [19] = 21.86, *p* = 0.29). Mei et al. (5) also found that meteorological factors do not affect NA (solar radiation [*r* = 0.006, *p* > 0.05]). In this study, most of the participants were interviewed after the experiments. They reported that they felt more relaxed after viewing scenery in the two weather conditions and did not experience much negative emotion. Therefore, there was no significant difference in NA scores between sunny and cloudy days.

In terms of emotional recovery, the data show that spaces with water bodies (space 1, 3, and 4) had stronger emotional recovery effects on cloudy days and that spaces without water bodies (space 2, 5, 6, 7) had stronger emotional recovery effects on sunny days. Thus, the presence of a water body was the main feature affecting emotional recovery under the two weather conditions, and emotional recovery effects due to viewing the water body were stronger on cloudy days. This contradicts the findings of White et al. (38), who argued that water bodies provide greater recovery under the sunny condition. Our hypothesis is that calm water is more likely to improve mood on cloudy days, and positive mood is more likely to be associated with flowing water on sunny days. The experimental season was conducted in autumn in Chengdu, during which time the water is relatively calm, and overall water flow is slow. From an evolutionary and circadian perspective, organisms are better adapted to being in quieter environments when the light is darker (65); thus, viewing serene water bodies may have a greater effect on emotional recovery on cloudy days. On sunny days, the restorativeness of the waterscape may come from experiencing light reflected off of the fluctuating water surface (66) and the sound of running water (20, 67). The calm river water in this study may have limited the restorative potential of spaces with water bodies, resulting in spaces without water bodies exhibiting greater emotional restorativeness on sunny days. The differences in restorative effects of spaces with and without water bodies under different weather conditions can be further explored in the future.

The perceived restoration effects of sunny and cloudy days were highly consistent. Under both weather conditions, higher PRS scores were observed in spaces with high naturalness (space 2, 4, 8, and 7), while lower PRS scores were observed in spaces with low naturalness (space 1, 3, 5, and 6). The data suggest that UGSs with high naturalness are perceived as more restorative under both weather conditions, a finding that is consistent with those of many previous studies (12, 13). Among the spaces, spaces 2 and 4, which had high naturalness and low SVF, exhibited greater perceived restoration under the two weather conditions. Previous studies have demonstrated that the amount of sky visible can enhance fascination, with a weak positive correlation observed between fascination and perceptual recovery (effects = 0.283, *p* < 0.01) (23, 68). This partially contradicts our findings. Our results showed that space 2 and 4 were rated as significantly higher in being away and high in fascination. We believe that this may be because lower SVF in a natural environment creates a sense of privacy, which can trigger daydreams that differ from daily work and life and unconsciously restore directed attention; the diversity and complexity of the natural environment may also promote fascination (67). The results of perceived restoration are consistent with the results of aesthetic preference, which supports a correlation between environment preference and perceived restoration (9, 10).

### 4.5. Combined physiological and psychological restoration

After a comprehensive observation of the combined physiological and psychological restoration effects across environments in two weather conditions, we found that space 1 (low naturalness, low SVF and with bodies of water) promoted physio-psychological restoration (physiological and emotional recovery) on cloudy days, and space 4 (high naturalness, low SVF and with bodies of water) promoted physio-psychological restoration (physiological, emotional and perceived restoration) on sunny days.

The results above show that an environment with a low SVF and bodies of water can promote the recovery of physiological and psychological health simultaneously in both weather conditions. Some possible explanations are that a lower SVF on sunny days is correlated with increased shading, while bodies of water increase the humidity. Together, these two factors improve microclimate comfort (24, 38), which is beneficial for relieving stress on sunny days and achieving physical and mental relaxation. In contrast to previous studies, White et al. (38) found that the effect of bodies of water on recovery on cloudy days was significantly lower than that on sunny days, possibly because areas with bodies of water have fewer opportunities to provide shelter in bad weather. However, combined with the results of this study, it may be proven that in cloudy environments that provide a low sense of security, a lower SVF increases the sense of enclosure and shelter of the environment (including bodies of water) as well as the greenery people are exposed to in UGSs (26). These two aspects could affect physio-psychological recovery by promoting physical activity (69), releasing tension (7) and aiding visitors in recovering the directed attention (6). Therefore, such environments also show good health recovery potential under cloudy conditions. The SVF results support previous findings that the restoration effect of green spaces does not always increase with the SVF (26, 46, 70). Although studies have shown that public physical and mental health generally improve with increasing SVF score (25, 71), our research may demonstrate that the restoration effect of UGSs does not always correlate negatively with the reduction in the SVF until it decreases to a certain threshold. Quantitative and in-depth research on SVF should be conducted in the future to assess the specific boundaries and characteristics of SVF that affect the restoration of UGSs.

### 4.6. Limitations

The limitations are as follows. Firstly, we chose completely cloudy and completely sunny days for the experiment. In future research, more weather types (e.g., partly cloudy) should be considered, and quantitative indicators such as illuminance and sun elevation angle should be incorporated. Additionally, each participant had to visit all 8 spaces and complete many questionnaires in 1 day in our study, which reduced the scientificity and reliability of the data. Moreover, the physiological status of participants may change from before to after lunch, which could have induced errors in the measurement of physiological indicators such as blood pressure. Finally, the study only focused on one park in Chengdu and conducted experiments in only one season. Perceptions of environmental temperature by participants may vary at different latitudes due to various factors such as adaptability. Perception of weather conditions might also be influenced by the season. Subsequent research should explore a broader range of experimental locations and seasons to enhance the universality of the research.

## 5. Conclusion

Until now, most studies have only focused on the influence of green space characteristics on restoration. This study innovatively broadened its scope by investigating weather factors (sunny and cloudy conditions), thus expanding the research domain of UGS restorative environments and introducing the notion of “comprehensive restorative environments.” This expansion offers a more scientifically grounded theoretical basis for the establishment and enhancement of restorative environments in diverse weather conditions across different regions. As evidenced by the findings, weather conditions do indeed impact restorative benefits. For instance, UGSs exhibit greater health recovery potential on sunny days compared to overcast ones, with females experiencing stronger psychological recovery benefits under both weather conditions. Moreover, there are also variations in restorative effects among different environmental types under two weather conditions. These conclusions provide new insights for research on health-supportive human habitats in the context of global climate change, offering nature-based solutions to meet the residents’ escalating demands of health restoration.

In future planning and design practices, UGSs in regions with frequent cloudy days should incorporate more water bodies with gentle flow and low gradient, as this proves to be an effective approach to enhance emotional restoration during overcast weather. UGSs in regions with frequent sunny days should consider increasing sky exposure while ensuring adequate shading to promote residents’ physical health. Additionally, sheltered environments that incorporate water bodies demonstrate the capacity to promote both physical and mental health restoration under both weather conditions, rendering them suitable for widespread implementation across various regions. Furthermore, the study also unveiled that the restorative benefits of water bodies might be influenced by factors other than weather, such as water flow conditions. Likewise, a more intricate relationship could potentially exist between sky openness and health restoration. Future research should delve deeper into these intriguing matters.

## Data availability statement

The datasets presented in this article are not readily available because protecting the privacy of the participants. Requests to access the datasets should be directed to XL, lixi@sicau.edu.cn.

## Ethics statement

The studies involving humans were approved by Ethics Committee of the College of Landscape Architecture, Sichuan Agricultural University, China. The studies were conducted in accordance with the local legislation and institutional requirements. The participants provided their written informed consent to participate in this study.

## Author contributions

SC: Conceptualization, Data curation, Formal analysis, Investigation, Methodology, Visualization, Writing – Original draft, Writing – Review & editing. ZS: Conceptualization, Investigation, Methodology, Writing – Review & editing. XL: Funding acquisition, Supervision, Writing – Review & editing. HL, LS, MJ, JD, EF, JM, NL, BG, XY, BL, JW: Writing – Review & editing.

## Funding

The authors declare financial support was received for the research, authorship, and/or publication of this article. This research was funded by the National Natural Science Foundation of China, grant number 31870703.

## Conflict of interest

The authors declare that the research was conducted in the absence of any commercial or financial relationships that could be construed as a potential conflict of interest.

## Publisher’s note

All claims expressed in this article are solely those of the authors and do not necessarily represent those of their affiliated organizations, or those of the publisher, the editors and the reviewers. Any product that may be evaluated in this article, or claim that may be made by its manufacturer, is not guaranteed or endorsed by the publisher.
