# Supplementary material for: Cloudy or sunny? Effects of different environmental types of urban green spaces on public physiological and psychological health under two weather conditions
Source: Front Public Health. 2023 Aug 28;11:1258848. doi: 10.3389/fpubh.2023.1258848 (PMC10493420; doi:10.3389/fpubh.2023.1258848)
Supplement: Supplementary file 1 [file Table_1.pdf]

Supplementary Material

Cloudy or sunny? Effects of different environmental types of urban green spaces on public physiological and psychological health under two weather conditions

Saixin Cao†, Zike Shang†, Xi Li*, Hao Luo, Lingxia Sun, Mingyan Jiang, Juan Du, Erkang Fu, Jun Ma, Nian Li, Baimeng Guo, Xiaofang Yu, Bingyang Lv and Jinde Wang

*** Correspondence:** Xi Li: lixi@sicau.edu.cn

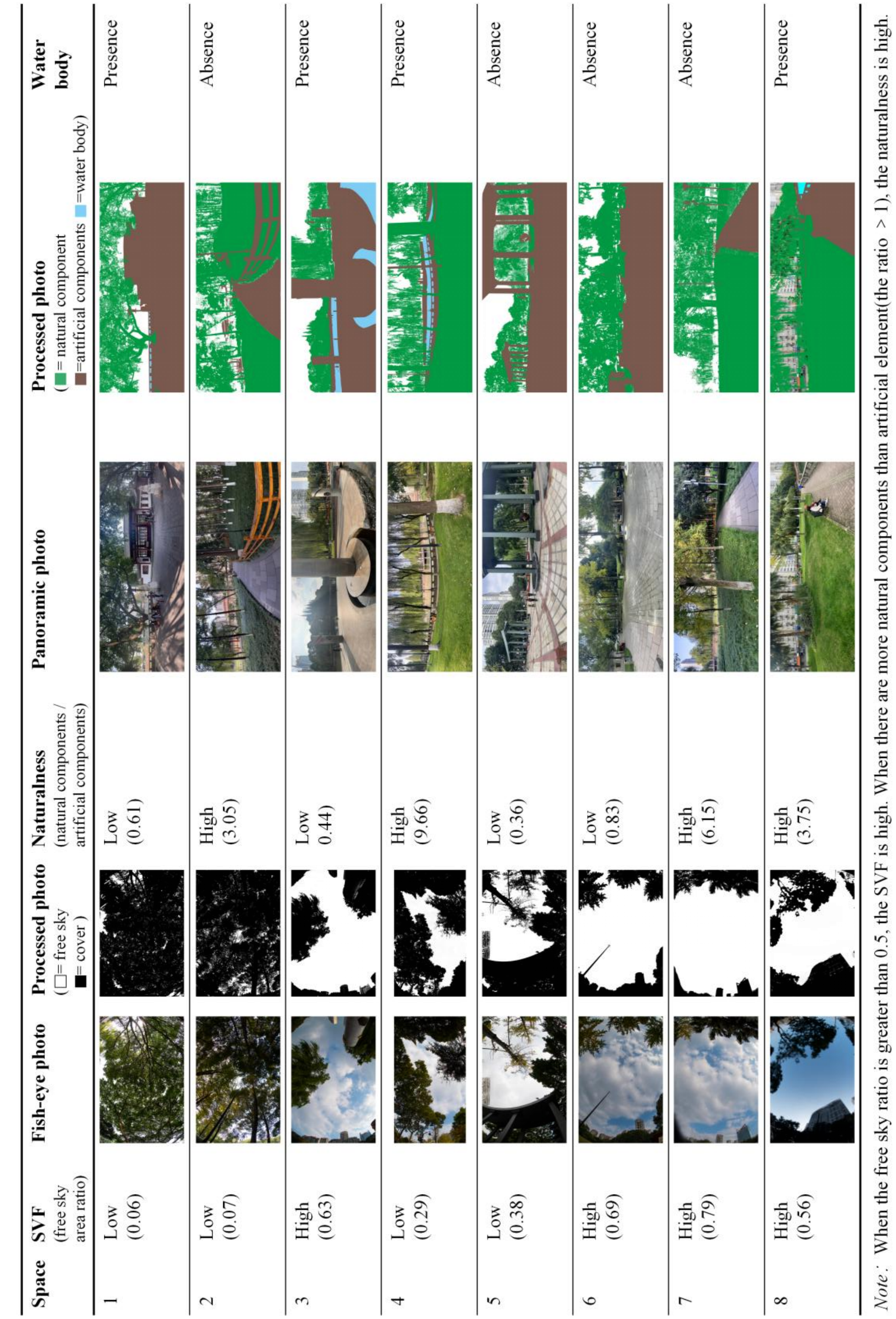

Figure S1. Specific environment features of the 8 spaces

Table S1. The Positive And Negative Affect Schedule (PANAS)

Name： Date： Space Number：

This scale consists of a number of words that describe different feelings and emotions. Please **CIRCLE THE NUMBER THAT BEST DESCRIBES HOW YOU FEEL RIGHT NOW**.

		Very slightly or not at all	A Little	Moderately	Quite a bit	Extremely
1.	interested	1	2	3	4	5
2.	distressed	1	2	3	4	5
3.	excited	1	2	3	4	5
4.	upset	1	2	3	4	5
5.	strong	1	2	3	4	5
6.	guilty	1	2	3	4	5
7.	scared	1	2	3	4	5
8.	hostile	1	2	3	4	5
9.	enthusiastic	1	2	3	4	5
10.	proud	1	2	3	4	5
11.	irritable	1	2	3	4	5
12.	alert	1	2	3	4	5
13.	ashamed	1	2	3	4	5
14.	inspired	1	2	3	4	5
15.	nervous	1	2	3	4	5
16.	determined	1	2	3	4	5
17.	attentive	1	2	3	4	5
18.	jittery	1	2	3	4	5
19.	active	1	2	3	4	5
20.	afraid	1	2	3	4	5

THANK YOU FOR YOUR COOPERATION

PLEASE BE SURE YOU HAVE ANSWERED EVERY ITEM

Table S2. Short-version revised Perceived Restorativeness Scale (PRS)

Short-version revised Perceived Restorativeness Scale (PRS)

Name： Date： Space Number：

Please complete the following questionnaire which is in regard to providing the restorative experience on a scale from 0 to 7, where 7 represented the highest positive rating and 1 the lowest rating. Please **CIRCLE THE NUMBER THAT BEST DESCRIBES HOW YOU FEEL RIGHT NOW.**

		**Restorative Experience**						
1.	Being here is an escape experience.	1	2	3	4	5	6	7
2.	Spending time here gives me a break from my day-to-day routine.	1	2	3	4	5	6	7
3.	It is a place to get away from it all.	1	2	3	4	5	6	7
4.	Being here helps me relax my tension.	1	2	3	4	5	6	7
5.	This place makes me feel free from work and daily life.	1	2	3	4	5	6	7
6.	The surrounding scenery here is in harmony.	1	2	3	4	5	6	7
7.	I'm quite curious about the unseen views in the scenery here.	1	2	3	4	5	6	7
8.	Coming here helps me extend a lot of good associations.	1	2	3	4	5	6	7
9.	The elements of the landscape are matched here .	1	2	3	4	5	6	7
10.	This place has fascinating qualities.	1	2	3	4	5	6	7
11.	There is much to explore and discover here.	1	2	3	4	5	6	7
12.	The setting is fascinating.	1	2	3	4	5	6	7
13.	I want to spend more time looking at the surroundings.	1	2	3	4	5	6	7
14.	I can do things I like here.	1	2	3	4	5	6	7
15.	Being here suits my personality	1	2	3	4	5	6	7
16.	I have a sense that I belong here.	1	2	3	4	5	6	7
17.	I can find ways to enjoy myself here.	1	2	3	4	5	6	7
18.	It is easy to find my way around here.	1	2	3	4	5	6	7

Table S3. Aesthetic Preference Questionnaire

Name： Date： Space Number：

Please answer the following two questions which are in regard to aesthetic preference on a scale from 0 to 5, where 5 represented the highest positive rating and 1 the lowest rating. Please **CIRCLE THE NUMBER THAT BEST DESCRIBES HOW YOU FEEL RIGHT NOW.**

1 Look at the sky. How would you rate the beauty of the sky at this moment? (1 2 3 4 5)

2 Look around. How would you rate the beauty of the environment at this moment? (1 2 3 4 5)
